# Specific human leukocyte antigen class I genotypes predict prognosis in resected pancreatic adenocarcinoma: a retrospective cohort study

**DOI:** 10.1097/JS9.0000000000000264

**Published:** 2023-04-07

**Authors:** Chenlei Wen, Lei Zhang, Ying Yang, Yangbing Jin, Dandan Ren, Zehui Zhang, Siyi Zou, Fanlu Li, Huaibo Sun, Jiabin Jin, Xiongxiong Lu, Junjie Xie, Dongfeng Cheng, Zhiwei Xu, Huan Chen, Beibei Mao, Jun Zhang, Jiancheng Wang, Xiaxing Deng, Chenghong Peng, Hongwei Li, Cen Jiang, Lin Lin, Henghui Zhang, Hao Chen, Baiyong Shen, Qian Zhan

**Affiliations:** a Department of General Surgery, Pancreatic Disease Center, Research Institute of Pancreatic Diseases, Ruijin Hospital, Shanghai Jiao Tong University School of Medicine; b Department of Laboratory Medicine, Ruijin Hospital, Shanghai Jiao Tong University School of Medicine; c State Key Laboratory of Oncogenes and Related Genes, National Research Center for Translational Medicine (Shanghai), Shanghai; d Genecast Biotechnology Co. Ltd, Wuxi, Jiangsu Province; e Biomedical Innovation Center, Beijing Shijitan Hospital, Capital Medical University, Beijing, China; f Beijing Key Laboratory for Therapeutic Cancer Vaccines, Beijing Shijitan Hospital, Capital Medical University, Beijing, China

**Keywords:** genotype, human leukocyte antigen class I, immunoediting, pancreatic adenocarcinoma, recurrence

## Abstract

Patients with resected pancreatic adenocarcinoma (PAAD) often experience short-term relapse and dismal survival, suggesting an urgent need to develop predictive and/or prognostic biomarkers for these populations. Given the potential associations of the human leukocyte antigen class I (*HLA*-I) genotype with oncogenic mutational profile and immunotherapy efficacy, we aimed to assess whether differential *HLA*-I genotype could predict the postoperative outcomes in resected PAAD patients.

**Materials and Methods:**

*HLA*-I (*A*, *B*, and *C*) genotyping and somatic variants of 608 Chinese PAAD patients were determined by targeted next-generation sequencing of matched blood cells and tumor tissues. *HLA*-*A*/*B* alleles were classified with the available definition of 12 supertypes. The Kaplan–Meier curves of disease-free survival (DFS) and multivariable Cox proportional-hazards regression analyses were performed to determine the survival difference in 226 selected patients with radical resection. Early-stage (I–II) patients constituted the majority (82%, 185/226) and some stage I–II individuals with high-quality tumor samples were analyzed by RNA-sequencing to examine immunophenotypes.

**Results:**

Patients with HLA-A02^+^B62^+^B44^−^ had significantly shorter DFS (median, 239 vs. 410 days; hazard ratio=1.65, *P*=0.0189) than patients without this genotype. Notably, stage I–II patients carrying HLA-A02^+^B62^+^B44^−^ had sharply shorter DFS than those without HLA-A02^+^B62^+^B44^−^ (median, 237 vs. 427 days; hazard ratio=1.85, *P*=0.007). Multivariate analysis revealed that HLA-A02^+^B62^+^B44^−^ was associated with significantly inferior DFS (*P*=0.014) in stage I–II patients but not in stage III patients. Mechanistically, HLA-A02^+^B62^+^B44^−^ patients were associated with a high rate of *KRAS* G12D and *TP53* mutations, lower *HLA-A* expression, and less inflamed T-cell infiltration.

**Conclusion:**

The current results suggest that a specific combination of germline HLA-A02/B62/B44 supertype, HLA-A02^+^B62^+^B44^−^, was a potential predictor for DFS in early-stage PAAD patients after surgery.

## Graphical abstract

**Figure:**
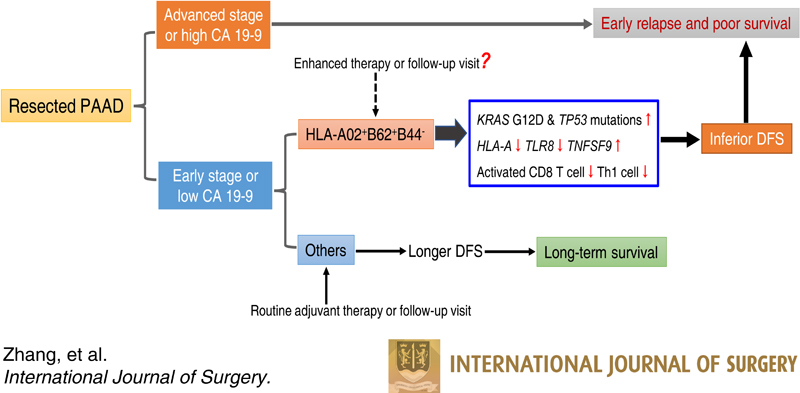

## Introduction

Highlights

- Specific human leukocyte antigen class I (*HLA*-I) genotypes negatively impact disease-free survival (DFS) in resected pancreatic adenocarcinoma (PAAD).
- HLA-A02^+^B62^+^B44^−^ correlates with inferior DFS in early-stage PAAD.
- Enriched driver mutations are associated with unfavorable *HLA*-I genotype.
- HLA-A02^+^B62^+^B44^−^ patients tend to have lower *HLA-A* expression.

PAAD is one of the most lethal causes of cancer mortality around the world. Although surgery is the curative treatment for most early-stage PAAD patients, the postsurgical recurrence rate within 1 year was above 50% and the 5-year survival rate was under 30%^1^. It is commonly recognized that large tumor size, lymph node involvement, high serum level of carbohydrate antigen 19-9 (CA 19-9) as well as main driver gene alterations (*KRAS*, *TP53*, *CDKN2A*, *SMAD4*) are associated with worse postoperative outcomes in PAAD patients^2–4^. The serum level of CA 19-9 is the only approved and extensively used noninvasive biomarker to monitor the disease recurrence after surgical resection. However, it is unclear why some early-stage (I–II) PAAD patients with normal CA 19-9 level (<37 U/ml) also experience short-term relapse and inferior postoperative survival, suggesting that there is an urgent need to develop robustly predictive and/or prognostic biomarkers for patients with resected PAAD.

*HLA*-I genotype-restricted immunoediting has been described in various solid tumors where recurrent oncogenic mutations tend to have poor HLA-I presentation^5^. It could shape the personalized mutational profile and mediate the immune escape during tumorigenesis via impairing antigen presentation in an oncogenic-dependent manner^5^. Given the abundant genetic polymorphism of *HLA* genes (http://hla.alleles.org/nomenclature/stats.html), it is hypothesized that each cancer patient carrying distinctive *HLA* alleles is gifted with a differentiated efficiency of antigen presentation, possibly resulting in different therapeutic response or clinical outcome. Previously, HLA-B44 or B62 supertype (clustered HLA molecules with similar antigen binding specificity) was reported to be associated with better or worse survival in melanoma patients following checkpoint blockade immunotherapies^6^. Nevertheless, whether *HLA*-I genotypes could influence postoperative outcomes in patients with early-stage cancers remains largely unknown.

In this study, we hypothesized that specific *HLA*-I genotypes could impact the disease recurrence for patients with early-stage PAAD after radical pancreatectomy. Herein, we analyzed somatic variants and the profiles of *HLA*-I (including *HLA*-*A*, *B*, and *C*) alleles and their supertypes in 608 PAAD patients with matched blood cells and carcinoma tissues. Then, 226 individuals undergoing radical pancreatectomy were selected to evaluate the associations between *HLA-*I genotypes and postoperative survival outcomes. We also performed data validation in an independent cohort of long-term survivors (survival >5 years after surgery) with PAAD versus those resected PAAD patients with early recurrence and multiple public cohorts of PAAD patients. Moreover, targeted transcriptomic sequencing of some high-quality PAAD tumor tissue samples was conducted to investigate the potential explanations for the associations between specific *HLA*-I genotypes and postoperative outcomes.

## Materials and methods

### Patient cohorts

**Figure 1:**
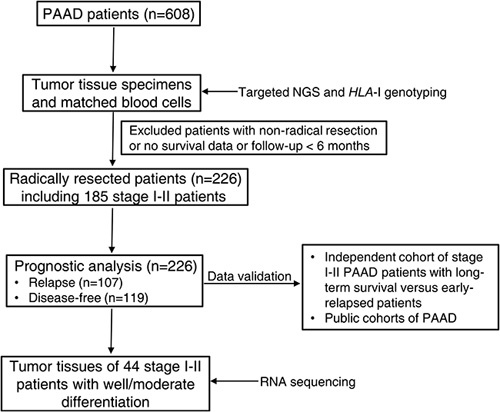
Flowchart of study design and patient selection in this study. *HLA*-I, human leukocyte antigen class I; NGS, next-generation sequencing; PAAD, pancreatic adenocarcinoma.

Our discovery cohort included 608 Chinese individuals pathologically diagnosed with PAAD from a genetic testing database or the Hospital between 14 March 2018, and 26 April 2020. All patients signed an informed consent form before this study. Among 608 patients, 226 of them (median follow-up of 336 days; range, 66–790 days) undergoing radical pancreatectomy (R0) in the hospital were selected for survival analyses of DFS and their clinicopathological parameters (including age, sex, histological grade, TNM stages, outcome data, etc.) were collected from this hospital. In this cohort (*N*=226), 202 patients (89.4%) received adjuvant therapy; the other 24 patients did not receive adjuvant therapy because of objective or subjective reasons, mainly due to age, low physical strength score, postoperative complications, poor nutrition status, or psychological factors. All adjuvant therapies followed the standard regimen (based on gemcitabine or 5-fluorouracil for 6 months) recommended in the guidelines, and there were no patients receiving neoadjuvant therapy or immunotherapy among them. The patients with nonradical resections or the postsurgical relapse-free individuals with follow-up less than 6 months were excluded from the survival analysis. DFS was defined as the time between surgical resection and disease relapse. The flowchart of patients’ selection is shown in Figure 1. In addition, an independent cohort of radically resected PAAD patients (stage I–II, *N*=42) with more than 5-year survival after surgery as well as some early-stage PAAD patients (*N*=78) with very short-term recurrence within 6 months after radical resection were used for validation. Cancer staging was based on the American Joint Committee on Cancer (AJCC) eighth edition of TNM staging system. This study (ethics approval number: 2013-70) was approved by the Medical Ethical Committee of the Hospital and performed in accordance with relevant guidelines. The work has been reported in line with the STROCSS criteria^7^ (Supplemental Digital Content 1, http://links.lww.com/JS9/A232) and was registered in ClinicalTrials.gov (NCT05483257, https://www.clinicaltrials.gov/ct2/show/NCT05483257?term=NCT05483257&draw=2&rank=1).

### Targeted next-generation sequencing

Tumor tissue specimens and matched peripheral blood cells underwent next-generation sequencing targeting 543 or 769 cancer-related genes (including *HLA-A*/*B*/*C*) at a CAP-certified laboratory or in the Hospital. DNA libraries were captured by HyperCap Target Enrichment Kit (Roche) and sequenced on the instruments of Illumina Novaseq. 6000 or NextSeq CN500. The average deduped sequencing depths of tissues and blood cells were ×830 and ×240, respectively. More details of sequencing and data analyses pipeline were described in the Supplementary Methods (Supplemental Digital Content 3, http://links.lww.com/JS9/A234).

### Germline *HLA*-I genotyping

Reads in regions of *HLA*-I (*A*, *B*, and *C*) genes were extracted from those BAM files of blood cells and then imported into software HLA-HD (v1.2.0.1)^8^ to identify *HLA-I* alleles with the following parameters: minimum_tag_size=50, rate_of_cutting=0.95. *HLA*-*A*/*B* alleles were classified with the available definition of supertypes like previously described^9^. Unsupervised clustering of HLA-A/B supertypes was performed using the Partitioning Around Medoid algorithm and distance was calculated via euclidean metric in the R package ConsensusClusterPlus. And R package ComplexHeatmap was used to display the clustering results.

### RNA sequencing

Expression levels of immune-related genes, signatures, or markers were determined by Oncomine Immune Response Research Assay (Cat. No. A32881; Thermo Fisher Scientific), an RNA-based next-generation sequencing assay targeting 395 genes like previously described^10^. RNA of tumor tissue specimens was extracted using truXTRAC extraction kit (Covaris). Quantification of isolated RNA was performed using Qubit RNA HS Assay Kit (Thermo Fisher Scientific) and then reversely transcribed into complementary DNA. RNA libraries were prepared by amplification with specific primers targeting 395 genes and sequenced on the Ion Torrent S5 Systems according to the manufacturer’s instructions. Gene expression was initially calculated as reads per million (RPM) and the normalization ratio determined by RPM of 10 housekeeping genes was used to normalize RPM counts for each gene in the examined samples. And the differential expression genes were identified through R package limma with a *P*-value less than 0.05 and abs(log_2_fold change) more than 1. The immune-related signatures or markers examined in this study were listed as follows: Activated CD8 T cell^11^, type 1 T helper (Th1) cell^11^, T-cell–inflamed gene expression profile (*CD2*, *CIITA*, *CXCL10*, *CXCL13*, *CXCR6*, *CD3E*, *GZMB*, *CD3D*, *HLA-DRA*, *HLA-E*, *IDO1*, *NKG7*, *IL2RG*, *LAG3*, *CCL5*, *STAT1*, *TAGAP*, *GZMK*)^12,13^, T-cell markers (*CD2*, *CD4*, *CD40LG*, *CD8B*, *CD3E*, *CD3D*, *CD8A*)^14^, IFN-γ signature (*CXCL10*, *CXCL9*, *HLA-DRA*, *IDO1*, *IFNG*, *STAT1*)^12^, Monocyte^11^. ssGSEA scores of these signatures were calculated by R package GSVA^15,16^.

### Statistical analyses

The Kaplan–Meier curves generated by GraphPad Prism 8.0 were used to estimate differences in DFS among grouped patients by a two-sided log-rank (Mantel–Cox) test. Stratified multivariable Cox proportional-hazards regression models were used to examine multivariable effects of identified germline HLA-A02/B62/B44 supertype combinations, clinical characteristics, and somatic alterations on DFS through the R packages survival and survminer. The statistical differences of above immune-related signatures or markers revealed in the boxplot were calculated by the R package ggpubr using the Wilcoxon test. The Spearman rank correlation test was used for assessing the correlations between HLA-A02/B62/B44 supertype combination or single supertype and common driver mutations or genetic alterations of oncogenic pathways. Fisher’s exact test or chi-square test was applied to compare the differences of categorical variables. All statistical tests were two-sided unless denoted otherwise. *P*-value less than 0.05 denoted statistical significance.

## Results

### Profiling of *HLA*-I genotype

**Figure 2:**
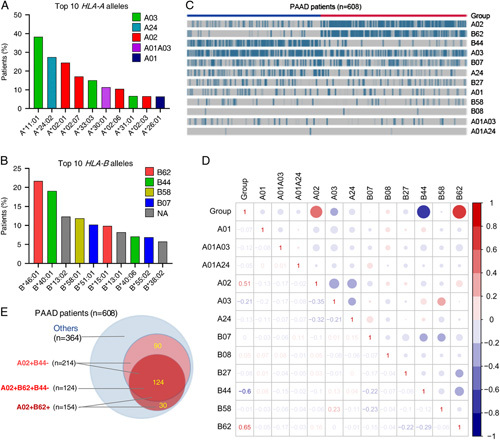
Landscape of *HLA*-I genotypes in Chinese PAAD patients. Population frequencies (%) of the top 10 germline *HLA-A* alleles (A) and *HLA-B* alleles (B) along with their corresponding supertypes in 608 PAAD patients. Unsupervised clustering (C) of HLA-A/B supertypes and matrix visualization (D) of the correlation between HLA supertypes and identified groups by unsupervised clustering in 608 PAAD patients. Positive correlations and negative correlations are shown in red or blue color. The degree of pairwise correlation with respect to Spearman’s correlation coefficient is displayed by the color intensity revealed as the size of the circle in the upper triangle or directly marked as the number in the lower triangle. (E) Venn diagram of the distribution of different HLA-A02/B62/B44 supertype combinations in 608 PAAD patients. *HLA*-I, human leukocyte antigen class I; NA, not available supertype; PAAD, pancreatic adenocarcinoma.

In total, 133 individual *HLA-A*, *HLA*-*B*, and *HLA*-*C* alleles (Supplementary Table S1, Supplemental Digital Content 2, http://links.lww.com/JS9/A233) were called in 608 PAAD patients, and 99 *HLA*-*A*/*B* alleles were assigned to 12 disparate supertypes with the available definition^9^, based on their peptide-anchor-binding specificity^9,17^. The top 10 *HLA-A*/*B* alleles and corresponding supertypes were listed in Figures 2A and B. Notably, clustering analysis revealed that the HLA-A02 supertype appeared to be co-existed with the HLA-B62 supertype (*P*=0.0001) in the same subgroup of patients, whereas HLA-B44 supertype was inclined to be mutually exclusive with A02 or B62 supertype (*P*=0.013 or 6.98e−13, respectively, Figs. 2C and D). Thus, these patients could be separated into one subgroup (HLA-A02^+^B62^+^B44^−^) by their innate combinations of HLA-A02/B62/B44 supertypes as depicted in Figure 2E.

### Identification of specific *HLA*-I genotypes and their impact on postoperative outcomes

**Figure 3:**
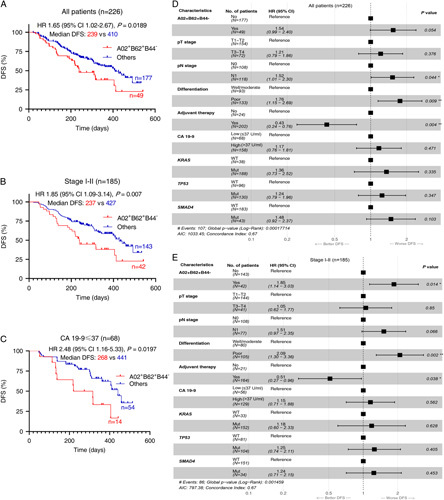
Impact of HLA-A02^+^B62^+^B44^−^ upon DFS of PAAD patients after radical resections. The Kaplan–Meier analyses of DFS by HLA-A02^+^B62^+^B44^−^ in all radically resected PAAD patients (*N*=226) (A) or stage I–II individuals (*N*=185) (B) or low CA 19-9 (≤37 U/ml) patients (*N*=68) (C). The *P*-value was calculated by a two-sided log-rank test. *P*<0.05 denoted statistical significance. Multivariable Cox proportional-hazards regression analysis of DFS demonstrated the unfavorable prognostic role of HLA-A02^+^B62^+^B44^−^ independent of clinical characteristics and driver mutations in all patients (*N*=226) (D) or stage I–II patients (*N*=185) (E). *P*<0.05 denoted statistical significance. Bars represent the 95% CI. TNM staging, American Joint Committee on Cancer (AJCC) eighth edition. CA 19-9, carbohydrate antigen 19-9; DFS, disease-free survival; HR, hazard ratio; Mut, mutant; PAAD, pancreatic adenocarcinoma; WT, wild type.

To reduce the potential bias produced by different surgical margin status affecting postoperative survival, we selected 226 individuals received radical pancreatectomy (R0) in a single center to perform Kaplan–Meier analyses. Their clinical characteristics were presented in Table 1. It is worth mentioning that early-stage (I–II) patients constituted the majority (82%, 185/226) in this cohort, which was distinguished from previously reported PAAD cohorts mainly including advanced-stage or metastatic patients. Besides, the profiles of *HLA*-*A*/*B*/*C* alleles and according to supertypes in 226 individuals were consistent with that observed in 608 patients (Supplementary Fig. S1A, Supplemental Digital Content 3, http://links.lww.com/JS9/A234, Table S1, Supplemental Digital Content 2, http://links.lww.com/JS9/A233). Intriguingly, patients with HLA-A02^+^B62^+^B44^−^ had significantly shorter DFS [median DFS, 239 vs. 410 days; hazard ratio (HR), 1.65; 95% CI, 1.02–2.67; *P*=0.0189] than patients without this genotype (Fig. 3A), while single of A02/B62/B44 supertype showed limited effect on DFS in these patients (Supplementary Table S2, Supplemental Digital Content 3, http://links.lww.com/JS9/A234). Importantly, stage I–II patients (*N*=185) carrying HLA-A02^+^B62^+^B44^−^ had sharply shorter DFS than other early-stage patients (median DFS, 237 vs. 427 days; HR, 1.85; 95% CI, 1.09–3.14; *P*=0.007) (Fig. 3B). Whereas this phenomenon was not observed in stage III patients (*N*=41) (Supplementary Fig. S1B, Supplemental Digital Content 3, http://links.lww.com/JS9/A234). The improved separation of survival curves in stage I–II individuals may be on account of the rationale that HLA-based immunoediting takes effect predominantly in the early phase of tumorigenesis^5^. It should be noted that the patients harboring B62^+^B44^−^ had obviously inferior survival compared with patients with B62^−^B44^+^ in A02-positive individuals (Supplementary Figs. S1C and D, Supplemental Digital Content 3, http://links.lww.com/JS9/A234). Furthermore, the significant difference of DFS between those patients with HLA-B44 supertype or not (median DFS, not reported vs. 410 days; HR, 0.33; 95% CI, 0.16–0.67; *P*=0.0154) was observed in extremely early-stage (IA–IB) individuals (*N*=86) (Supplementary Fig. S1E, Supplemental Digital Content 3, http://links.lww.com/JS9/A234). It seemed that B44 supertype was likely a favorable prognostic factor for early PAAD and B62 supertype was the opposite, which was akin to the positive or negative effect of B44 or B62 supertype on survival in melanoma cohorts with checkpoint blockade immunotherapy^6^. But these two supertypes were almost required to be combined with A02 supertype to reach the statistical significance for univariate survival analyses in the whole cohort and some subgroup (Supplementary Table S3, Supplemental Digital Content 4, http://links.lww.com/JS9/A235).

**Table 1:** Clinical characteristics of pancreatic adenocarcinoma patients (*N*=226) with radical pancreatectomy

	*n* (%)			
Characteristics	All patients (*N*=226)	A02^+^B62^+^B44^−^ (*n*=49)	Others (*n*=177)	*P*
Sex				0.1
Male	141 (62.4)	36 (73.5)	105 (59.3)	
Female	85 (37.6)	13 (26.5)	72 (40.7)	
Age (years)				0.889
≤65	148 (65.5)	115 (65.0)	33 (67.3)	
>65	78 (34.5)	62 (35.0)	16 (32.7)	
pT stage				0.36
T1–T2	154 (68.1)	30 (61.2)	124 (70.1)	
T3	44 (19.5)	13 (26.5)	31 (17.5)	
T4	28 (12.4)	6 (12.2)	22 (12.4)	
pN stage				0.767
N0	108 (47.8)	22 (44.9)	86 (48.6)	
N1	118 (52.2)	27 (55.1)	91 (51.4)	
AJCC staging				0.441
IA	26 (11.5)	7 (14.3)	19 (10.7)	
IB	60 (26.5)	9 (18.4)	51 (28.8)	
IIA	22 (9.7)	6 (12.2)	16 (9.0)	
IIB	77 (34.1)	20 (40.8)	57 (32.2)	
III	41 (18.1)	7 (14.3)	34 (19.2)	
Differentiation				0.274
Well/moderate	93 (41.2)	24 (49.0)	69 (39.0)	
Poor	133 (58.8)	25 (51.0)	108 (61.0)	
CA 19-9 level (U/ml)				0.932
≤37	68 (30.1)	14 (28.6)	54 (30.5)	
>37	158 (69.9)	35 (71.4)	123 (69.5)	
Adjuvant therapy				0.497
No	24 (10.6)	7 (14.3)	17 (9.6)	
Yes	202 (89.4)	42 (85.7)	160 (90.4)	

TNM staging, AJCC eighth edition.

Two-sided chi-square test, *P*<0.05 denoted statistical significance.

AJCC, American Joint Committee on Cancer; CA 19-9, carbohydrate antigen 19-9.

Notably, HLA-A02^+^B62^+^B44^−^ could help to identify patients with worse outcomes (median DFS, 268 vs. 441 days; HR, 2.44; 95% CI, 0.94–6.31; *P*=0.0157) (Fig. 3C) among patients (*N*=68) with preoperative normal CA 19-9 (≤37 U/ml) who are generally believed to possess relatively better prognosis than their counterparts, whereas there was no significant statistical difference on DFS between HLA-A02^+^B62^+^B44^−^ or not in high-CA 19-9 (>37 U/ml) patients (*N*=158) (Supplementary Fig. S1F, Supplemental Digital Content 3, http://links.lww.com/JS9/A234). In addition, stage I–II patients with HLA-A02^+^B62^+^B44^−^ and preoperative high level of CA 19-9 had a higher 6-month relapse rate than those with a low level of CA 19-9 and without HLA-A02^+^B62^+^B44^−^ (32 vs. 14%, *P*=0.0745) (Supplementary Fig. S1G, Supplemental Digital Content 3, http://links.lww.com/JS9/A234).

### Multivariate analysis

Then, we performed multivariable Cox proportional-hazards regression analyses (adjustment for clinicopathologic characteristics, adjuvant therapy, CA 19-9 level, and driver mutations) to further explore the independent prognostic role of HLA-A02^+^B62^+^B44^−^. Among all patients, HLA-A02^+^B62^+^B44^−^ was a potential risk factor with marginal statistical significance (HR, 1.54; 95% CI, 0.99–2.40; *P*=0.054), while pN stage, differentiation, and adjuvant therapy had a significant impact on DFS (Fig. 3D). In patients with stage I–II PAAD, multivariate analysis suggested that HLA-A02^+^B62^+^B44^−^ was associated with significantly inferior DFS (HR, 1.85; 95% CI, 1.14–3.03; *P*=0.014) (Fig. 3E) but not in stage III patients (Supplementary Fig. S2, Supplemental Digital Content 3, http://links.lww.com/JS9/A234). Moreover, HLA-A02^+^B62^+^B44^−^ was associated with shorter DFS in patients with low level of CA 19-9 (HR, 2.09; 95% CI, 0.90–4.9; *P*=0.086), which was superior to other prognostic factors (Supplementary Figs. S3 and S4, Supplemental Digital Content 3, http://links.lww.com/JS9/A234). Collectively, HLA-A02^+^B62^+^B44^−^ was a potential predictor of disease recurrence in PAAD patients after radical surgeries, especially in patients with early-stage or low CA 19-9 level.

To validate the predictive value of HLA-A02^+^B62^+^B44^−^, we performed germline *HLA*-I genotyping of two independent cohorts: cohort 1 included stage I–II PAAD patients (*N*=42) with radical pancreatectomy and survived over 5 years since surgery (long-term survival more than 5 years, LTS5 cohort); cohort 2 included early-stage PAAD patients (*N*=78) who relapsed within 6 months (early recurrence in 6 months, ER6 cohort) after radical surgery. In ER6 cohort, there were 39 patients with postoperative relapse in 3 months (early recurrence in 3 months, ER3 cohort) and 39 relapsed during 3–6 months after surgery. Patients in ER3 cohort had higher rate of HLA-A02^+^B62^+^B44^−^ or A02 than those in LTS5 cohort (51.28 vs. 30.95%, *P*=0.331; 69.23 vs. 50%, *P*=0.078) (Supplementary Table S4, Supplemental Digital Content 3, http://links.lww.com/JS9/A234). Importantly, a clear step-down of the patients carrying HLA-A02^+^B62^+^B44^−^ or A02 supertype was observed from ER3/6 (early recurrence in 3 or 6 months) cohorts towards LTS5/6/7/8 (long-term survival more than 5, 6, 7, or 8 years) cohorts in which there was only one individual with A02^+^B62^+^B44^−^ in 15 patients surviving more than 8 years (Supplementary Fig. S5A, Supplemental Digital Content 3, http://links.lww.com/JS9/A234). In addition, there was a significant decrease of the frequency of A02 supertype among ER3 cohort, LTS7 cohort, and those patients with a survival of 5–7 years (*P*=0.0329, chi-square test for trend) (Supplementary Fig. S5B, Supplemental Digital Content 3, http://links.lww.com/JS9/A234), suggesting that specific unfavorable *HLA*-I genotypes may be negatively correlated with long-term survival of PAAD patients, particularly in those very long-term survivors (VLTSs).

### Potential explanations for impact of *HLA*-I genotype on prognosis

**Figure 4:**
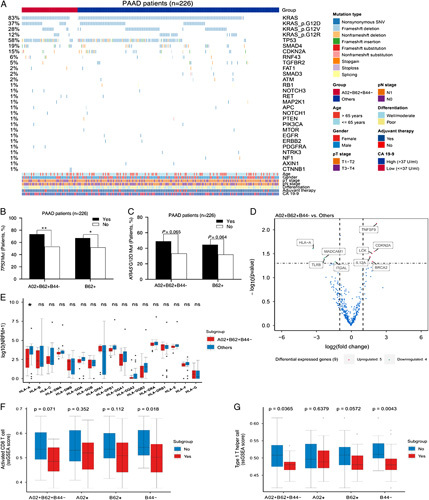
HLA-I-associated mutational profiles and characterized immunophenotype in radically resected PAAD. (A) Comparison of mutational landscape between HLA-A02^+^B62^+^B44^−^ patients versus others in 226 PAAD patients undergoing radical resections. Enrichment of *TP53* mutations (B) or *KRAS* G12D mutations (C) in those patients characterized by HLA-A02^+^B62^+^B44^−^ versus others without this genotype in 226 patients. The *P*-value was calculated by two-sided Fisher’s exact test. *P*<0.05 denoted statistical significance. *P<0.05; **P<0.01. (D) Identification of the genes with differential expression through R package limma with *P*<0.05 and abs(log_2_fold change) more than 1 between HLA-A02^+^B62^+^B44^−^ individuals versus others in stage I–II PAAD patients (*N*=44) with well/moderate differentiation. (E) A significant downregulation of *HLA-A* in tumor tissues of the individuals with HLA-A02^+^B62^+^B44^−^ versus others in stage I–II PAAD patients (*N*=44) with well/moderate differentiation. The *P*-value was calculated by a two-sided Wilcoxon test. *P*<0.05 denoted statistical significance. *P<0.05; **P<0.01. Decreased expression levels of Activated CD8 T cell (F) and type 1 T helper cell (G) in tumor tissues of the individuals with HLA-A02^+^B62^+^B44^−^ or single A02/B62/B44 supertype versus others in stage I–II PAAD patients (*N*=44) with well/moderate differentiation. The *P*-value was calculated by a two-sided Wilcoxon test. *P*<0.05 denoted statistical significance. Boxplots indicate the median, the first and third quartiles. Bars represent 1.5 times the interquartile range and outlying points are plotted individually. TNM staging, American Joint Committee on Cancer (AJCC) eighth edition. *HLA*-I, human leukocyte antigen class I; ns, not significant; PAAD, pancreatic adenocarcinoma.

To address why HLA-A02^+^B62^+^B44^−^ was associated with poor DFS in radically resected PAAD patients, we examined potential causes from two respects: mutational feature and gene expression. Given the generally low presentation of oncogenic alterations by HLA-I molecules^5,18^, we suspected that certain driver mutations might be further enriched in those individuals carrying unfavorable *HLA*-I genotypes. In effect, HLA-A02^+^B62^+^B44^−^ and single B62 supertype were significantly correlated with *TP53* mutations and/or *KRAS* G12D mutations (Figs. 4A–C, Supplementary Fig. S6A, Supplemental Digital Content 3, http://links.lww.com/JS9/A234), while no statistical correlations with other major mutated driver genes (*CDKN2A*, *SMAD4*) or hotspot mutations (*KRAS* G12V/G12R) were observed in this analysis (Supplementary Fig. S6A, Supplemental Digital Content 3, http://links.lww.com/JS9/A234). In addition, the alterations of p53 pathway and/or Wnt pathway of 10 vital oncogenic signaling pathways^19^ were statistically associated with specific *HLA*-I genotypes (Supplementary Fig. S6B, Supplemental Digital Content 3, http://links.lww.com/JS9/A234). Considering the association of alterations in *KRAS* and *TP53* with worse outcomes of resected PAAD patients like previously described^4,20^, reduced DFS of the individuals carrying HLA-A02^+^B62^+^B44^−^ could be partly attributed to their augmented *KRAS* G12D and *TP53* mutations. Notwithstanding, the A02 supertype alone was not found to be linked to any alterations of main driver genes or cancer-related pathways (Supplementary Figs. S6A and B, Supplemental Digital Content 3, http://links.lww.com/JS9/A234).

Next, some stage I–II individuals (*N*=44) with available tissue specimens and well/moderate differentiation were selected from 226 PAAD patients to determine whether various *HLA*-I genotypes were implicated in differential immunophenotype of tumor microenvironment by RNA-sequencing^10^. As shown in Figure 4D, several significantly downregulated or upregulated genes between HLA-A02^+^B62^+^B44^−^ (*N*=14) and others (*N*=30) were identified. Notably, the expression of *HLA-A* was markedly reduced in tumor tissues of HLA-A02^+^B62^+^B44^−^ patients or A02-positive individuals relative to other cases (Figs. 4D and E, Supplementary Figs. S7A and B, Supplemental Digital Content 3, http://links.lww.com/JS9/A234), whereas there were scarcely any similar observations of other *HLA* encoding genes. Except for the A02 supertype, such dropped expression of *HLA-A* was absent between the patients with any other HLA-A supertypes or not. Even the individuals with HLA-A03 supertype had dramatically higher expression of *HLA-A* than that of those HLA-A03-negative individuals (*P*<0.001) (Supplementary Figs. S7C and D, Supplemental Digital Content 3, http://links.lww.com/JS9/A234). That is to say, the PAAD patients carrying HLA-A02 supertype rather than other HLA-A supertypes appeared to be more prone to have downregulated *HLA-A*, consequently facilitating immune evasion owing to lessened expression of HLA and impaired antigen presentation in cancer cells^21–23^, which was likely a contributory cause for short-term recurrence of HLA-A02^+^B62^+^B44^−^ individuals infrequently observed in PAAD LTSs. Besides, *HLA-B*/*C* expression was obviously decreased in B44-negative versus B44-positive individuals or B62-positive versus B62-negative individuals (Supplementary Figs. S7E and F, Supplemental Digital Content 3, http://links.lww.com/JS9/A234). However, no marked difference of *HLA-B*/*C* expression was observed between HLA-A02^+^B62^+^B44^−^ patients and other patients (Figs. 4D and E), implying that reduction of *HLA-A* but not *HLA-B*/*C* might be more relevant to worse prognosis of HLA-A02^+^B62^+^B44^−^ individuals.

In addition to *HLA-A*, there was a significantly lower expression of *TLR8* (toll-like receptor 8) in HLA-A02^+^B62^+^B44^−^ patients than in other patients, whereas a significantly higher of *TNFSF9* (tumor necrosis factor ligand superfamily member 9) was present in the former than in the latter (Fig. 4D). In consistent with the differential expression of *TLR8* and *TNFSF9* between HLA-A02^+^B62^+^B44^−^ and others, the significant downregulation of *TLR8* or upregulation of *TNFSF9* was also observed in B62-positive versus B62-negative individuals (Supplementary Fig. S7E, Supplemental Digital Content 3, http://links.lww.com/JS9/A234) or B44-negative versus B44-positive individuals (Supplementary Fig. S7F, Supplemental Digital Content 3, http://links.lww.com/JS9/A234), respectively. Likewise, the expression levels of Activated CD8 T cell, Th1 cell, and other immune-related signatures or markers (T-cell– inflamed gene expression profile, T-cell markers, etc.) exhibited a significant or considerable reduction in HLA-A02^+^B62^+^B44^−^ or B44-negative patients compared to their counterparts (Figs. 4F and G, Supplementary Figs. S8A–D, Supplemental Digital Content 3, http://links.lww.com/JS9/A234). Accordingly, such unfavorable immunophenotype was likely more difficult to control cancer cells and eventually bring about poorer outcomes of the individuals with HLA-A02^+^B62^+^B44^−^ relative to other individuals.

### Data validation

Finally, we performed validation of our findings in several public cohorts of PAAD. Given the ethnic difference of HLA-I supertypes between Chinese versus Western populations^17^ and without *HLA*-I genotyping data in TCGA (The Cancer Genome Atlas) or ICGC (International Cancer Genome Consortium), co-mutant *KRAS* G12D and *TP53* correlated with HLA-A02^+^B62^+^B44^−^ was used as a derived genomic ‘indicator’ to test the prognostic significance of HLA-A02^+^B62^+^B44^−^ for PAAD in public data. We found that PAAD patients with concomitant *KRAS* G12D and *TP53* mutations indeed had even worse overall survival (OS) compared with other PAAD patients in both entire TCGA cohort (*N*=179) (median OS, 15.12 vs. 21.44 months; HR, 2.01; 95% CI, 1.19–3.39; *P*=0.0013) and stage I–II PAAD patients (*N*=167) of TCGA cohort (median OS, 15.12 vs. 20.84 months; HR, 1.88; 95% CI, 1.11–3.20; *P*=0.0048) (Supplementary Figs. S9A and B, Supplemental Digital Content 3, http://links.lww.com/JS9/A234). Likewise, the PAAD patients with *KRAS* G12D plus *TP53* co-mutations had much shorter OS (median OS, 369 vs. 633 days; HR, 1.81; 95% CI, 1.28–2.56; *P*=0.0001) than that of other PAAD patients in ICGC cohort (*N*=235) (Supplementary Fig. S9C, Supplemental Digital Content 3, http://links.lww.com/JS9/A234). Moreover, the prognostic effect of *KRAS* G12D and *TP53* co-mutations was further strengthened (median OS, 361.5 vs. 730 days; HR, 2.05; 95% CI, 1.35–3.13; *P*<0.0001) in stage I–II patients (*N*=160) of ICGC cohort (Supplementary Fig. S9D, Supplemental Digital Content 3, http://links.lww.com/JS9/A234). Apparently, it resembled our observed enhanced impact of HLA-A02^+^B62^+^B44^−^ on DFS in early-stage PAAD patients relative to all patients (Figs. 3A and B). Instead, there was barely any difference (*P*=0.6427) of OS between the patients with co-occurrence of *KRAS* G12D and *TP53* mutations or not in a metastatic PAAD cohort^24^ (*N*=293) (Supplementary Fig. S9E, Supplemental Digital Content 3, http://links.lww.com/JS9/A234), which was similar to our observation that HLA-A02^+^B62^+^B44^−^ failed to stratify in stage III patients with PAAD (Supplementary Fig. S1B, Supplemental Digital Content 3, http://links.lww.com/JS9/A234). Briefly, in accordance with the stage-specific influence of HLA-A02^+^B62^+^B44^−^ on DFS in our cohort, its ‘indicator’ (co-mutant *KRAS* G12D and *TP53*) also had associations with poor survival of only early-stage but not late-stage PAAD patients in public cohorts.

## Discussion

In this study, we identified the specific *HLA*-I genotype, HLA-A02^+^B62^+^B44^−^, as a potential prognostic factor for DFS in PAAD patients with radical resections, especially in those stage I–II or low CA 19-9 (≤37 U/ml) individuals. Moreover, we noticed that HLA-A02^+^B62^+^B44^−^ appeared to be negatively correlated with long-term survival in an independent cohort of PAAD LTSs (surviving more than 5 years since surgery) and early-stage PAAD patients with early relapse in 3 or 6 months. Correspondingly, HLA-A02^+^B62^+^B44^−^-related ‘indicator’ co-occurrence of *KRAS* G12D and *TP53* mutations was verified to be significantly relevant to poor outcomes in stage I–II PAAD patients in both TCGA and ICGC cohort. Mechanistically, tumor with HLA-A02^+^B62^+^B44^−^ seemed to be associated with increased *KRAS* G12D and *TP53* mutations, attenuated antigen presentation, and antitumor immune response.

According to recent NCCN guideline for PAAD, surveillance via radiological examination or CA 19-9 level should be performed every 3–6 months for 2 years after surgery, and adjuvant therapy is recommended for all patients unless they have poor performance status. Even so, postoperative relapse within one year and miserable outcomes are commonly found in stage I–II PAAD patients with low CA 19-9. How to identify certain individuals subject to recurrence remains troublesome for clinical management of resected PAAD. Some studies revealed the promising prognostic significance of perioperative circulating tumor DNA (ctDNA) superior to imaging or CA 19-9 results for PAAD^25–28^. However, the extremely low abundance of ctDNA might hinder the application of ctDNA-based liquid biopsies in PAAD patients with radical resections. In comparison with ctDNA detection, *HLA*-I genotyping based on peripheral blood cells is easier to implement. Importantly, our data showed the relevance of HLA-A02^+^B62^+^B44^−^ to poor DFS in early-stage, low CA 19-9, or well/moderate-differentiated PAAD patients. Perhaps enhanced therapy or follow-up visit rather than routine adjuvant therapy or follow-up visit is needed for the PAAD patients with HLA-A02^+^B62^+^B44^−^. In other words, *HLA*-I genotyping may be conductive to clinical decision-making of those resected PAAD patients with low risk evaluated by current prognostic parameters (early stage, low CA 19-9, well/moderate differentiation, etc.).

Prior studies reported the promising value of a single HLA-B62 or B44 supertype in predicting the efficacy of immunotherapy in melanoma^6^ and the prognostic role of HLA-A02* genotype (not A02 supertype) in epithelial ovarian cancer^29^, while the clinical significance of HLA-I supertype combinations in cancer patients is overlooked to a large extent so far. To our knowledge, this is the first study to investigate the impact of inherent HLA-A02/B62/B44 supertype combination on survival outcomes in patients with PAAD. The results demonstrated the potential meaning of *HLA* genetic background combined with clinical characteristics for predicting the prognosis of resected PAAD. It is noteworthy that both HLA-A02^+^B62^+^B44^−^ and its ‘indicator’ *KRAS* G12D and *TP53* co-mutations failed to exhibit prognostic significance in advanced or metastatic PAAD patients of our cohort or public cohort, which is likely owing to the widespread immunosuppression and ineffective immunoediting in late-stage PAAD than in early-stage PAAD^5,30^. Therefore, the alterations of major driver genes are perhaps not suitable to be used as universal biomarkers for evaluating outcomes of those PAAD individuals undergoing radical (mainly for early-stage individuals) or nonradical (mainly for late-stage individuals) resections like previously performed^4^. Many studies have shown the associations of alterations in four established driver genes (*KRAS*, *TP53*, *CDKN2A*, *SMAD4*) with clinical outcomes of resected PAAD patients^4,20,31^, whereas there seems to be no consensus about the well-defined concurrent mutations of driver genes (e.g. *KRAS* G12D and *TP53* co-mutations) as prognostic biomarkers for stage-specific patients and it could be expanded by our observations. Enriched *KRAS* G12D and *TP53* mutations were significantly correlated with HLA-B62 supertype but not A02 or non-B44 supertype in our results, indicating that potential neoantigens derived from these driver mutations might be more difficult to be presented in B62-positive patients than in others, which is in agreement with restrained neoantigen recognition of B62 supertype owing to innate structural features as shown by molecular dynamics stimulations^6^. More details of the interaction between various oncogenic alterations of PAAD and diverse *HLA*-I genotypes in different ethnic populations as well as their impact on prognosis remain to be elucidated in future studies.

Postsurgical recurrence is a hard nut to crack for most solid tumors and is particularly deadly in resected PAAD patients with just 23-month median OS and 13-month median DFS after pancreatectomy^4,32,33^. Poor survival is closely related with typical early recurrence in PAAD patients following surgeries; nonetheless, why some patients with stage I–II PAAD received radical resections relapse shortly is still poorly understood. In principle, tumor relapse is mainly determined by two aspects: oncogenic genomic alterations of residual disease after surgical removal and antitumor response of individualized host immune system closely connected with *HLA* polymorphism. In addition to enrichment of certain driver mutations in HLA-A02^+^B62^+^B44^−^ patients, special immunophenotype characterized by downregulation of *HLA-A*/*TLR8* and upregulation of *TNFSF9* was observed in these patients (Fig. 4D). It is worth mentioning that high expression level of *TNFSF9* has been recently reported to be significantly associated with poor survival and negatively correlated with CD8 T-cell infiltration in public datasets of PAAD^34^. Moreover, despite a lack of understanding about the prognostic role of *TLR8* in PAAD, activation of TLR8 signaling has been widely believed to inhibit regulatory T cells (Tregs) function and enhance antitumor immunity^35–37^. In view of the prevalent Tregs-inducing immunosuppressive microenvironment in PAAD^38–40^, it makes sense that declining expression of *TLR8* could aggravate the immunosuppression state due to Tregs and impede antitumor immune response by CD8 T cells and Th1 cells in HLA-A02^+^B62^+^B44^−^ or B62-positive patients as shown in our results (Figs. 4F and G). In short, A02 supertype combined with B62-positivity and B44-negativity probably co-participated in contributing to the unfavorable immune contexture for recurrent PAAD involved in depressed expression of *HLA-A*/*TLR8*, and increased *TNFSF9* expression along with weakened antitumor immunity. Given the potential of TLR8 and TNFSF9 as new targets for cancer immunotherapy^36,41^, how specific *HLA*-I genotypes influence their expression in tumor microenvironment and the role of HLA-A02^+^B62^+^B44^−^ in treatment outcomes of related drugs targeting TLR8 or TNFSF9 deserve to be further studied.

Neoantigen-mediated immune response has been shown to be correlated with long-term survival of resected pancreatic cancer^42^, though this malignancy is generally considered to have an immune-excluded tumor milieu with a lack of efficiently infiltrated CD8 T cells^43^. Effective antitumor immune response derived from neoantigens relies on successful antigen presentation by HLA molecules at two critical events: T-cell priming initiated by dendritic cells taking up neoantigens and T-cell killing of targeted tumor cells with tumor-specific antigens being presented on cell surface^23,44^. In terms of mechanism, qualitative or quantitative changes (reduced expression, somatic mutation, loss of heterozygosity, etc.) of HLA-I likely lead to tumor immune evasion and immunotherapy failure^21–23^. Accordingly, based upon marked downregulation of *HLA-A* in A02-positive individuals versus A02-negative individuals as noted above, A02 supertype seems to facilitate short-term relapse in PAAD patients after surgery but hamper their long-term survival benefit. Indeed, it is in accordance with our observations that PAAD LTSs or VLTSs had even lower frequencies of A02 supertype or HLA-A02^+^B62^+^B44^−^ than in those stage-matched PAAD patients with early recurrence. Typically, there were no A02 supertype alleles identified in a rare PAAD VLTS who carried HLA-A*11:01 allele of A03 supertype had survived over 14 years in the context of multiple surgeries and metastases across one decade^45^. More importantly, her primary and metastatic tumors were always governed by *KRAS* G12V mutations predicted to be neoantigens with strong affinity to HLA-A*11:01, while she did not have any other common immune-silent driver mutations in PAAD^45^. Therefore, it could be supposed to infer that specific neoantigens in combination with favorable *HLA*-I genotypes, but neither alone, bring about good postoperative outcomes of certain PAAD patients, which might provide enlightenment for therapeutic neoantigen vaccine development in cancer patients.

While we provide considerable evidence supporting the prognostic value of specific *HLA*-I genotypes for resected PAAD, there are still some limitations. Due to no independent cohort of resected PAAD patients with enough *HLA*-I genotyping information and survival data, we fail to directly validate our observations in another PAAD cohort, though indirect validation results of HLA-A02^+^B62^+^B44^−^ in public datasets of PAAD are in line with expectations. Besides, examination of tumor immune microenvironment by RNA sequencing was performed in just 44 patients with high-quality tissue specimens and protein data of tumor-infiltrating lymphocytes of this cohort is absent. Multiplex immunohistochemistry may further reveal and verify the associations of specific *HLA*-I genotypes with tumor immune microenvironment.

## Conclusions

In summary, the current study suggested that specific combination of germline HLA-A02/B62/B44 supertype, HLA-A02^+^B62^+^B44^−^, was a potential predictor for DFS in PAAD patients after surgery, especially in stage I–II or low CA 19-9 (≤37 U/ml) patients. Mechanistically, patients with HLA-A02^+^B62^+^B44^−^ was associated with high rate of *KRAS* G12D and *TP53* mutations, lower *HLA-A* expression, and less inflamed T-cell infiltration. Together, a comprehensive assessment of the battle between driving tumor cells versus personalized immune system other than focusing on one side of the matter can better appreciate interindividual survival differences in resected PAAD.

## Ethical approval and consent to participate

This study was approved by the Medical Ethical Committee of the Hospital and performed in accordance with relevant guidelines (ethics approval number: 2013-70). All patients signed an informed consent form before this study.

## Sources of funding

This work was supported by grant from the National Natural Science Foundation of China (No. 81502695, 81672325, 81871906, 82073326) and the Shanghai Sailing Program (No. 20YF1426900). Besides, this study was granted by the National Key Sci-Tech Special Project of China (No. 2018ZX10302207).

## Author contribution

L.Z., Q.Z., and C.W.: conception and design. B.S., Ha.C., H.Z., and L.L.: project supervision. Y.Y., D.R., L.Z., H.S., Hu.C., and B.M.:bioinformatic and statistical analysis. L.Z., Q.Z., C.W., and Y.Y.: data mining and interpretation. Y.Y., D.R., and L.Z.: figures. Y.J., Z.Z., S.Z., F.L., J.J., X.L., J.X., D.C., and Z.X.: selection and collection of clinical samples and clinical data. J.Z., J.W., X.D., C.P., H.L., C.J., Hu.C., and B.M.: constructive advising and discussion. L.Z., Y.Y., Q.Z., C.W., B.S., Ha.C., H.Z., and L.L.: manuscript writing and editing. All authors have read and approved the final manuscript.

## Conflicts of interest disclosure

The authors declare that they have no financial conflict of interest with regard to the content of this report.

## Research registration unique identifying number (UIN)

1. Name of the registry: Clinicaltrials.gov
2. Unique Identifying number or registration ID: NCT05483257
3. Hyperlink to your specific registration (must be publicly accessible and will be checked): https://www.clinicaltrials.gov/ct2/show/NCT05483257?term=NCT05483257&draw=2&rank=1

## Guarantors

Baiyong Shen and Henghui Zhang.

## Data availability statement

The public datasets from prior studies are available at the following accession numbers: TCGA-PAAD, cBioPortal for Cancer Genomics at: http://cbioportal.org/msk-impact; ICGC-PAAD, https://dcc.icgc.org/projects/PACA-CA; Metastatic PAAD cohort, cBioPortal for Cancer Genomics at: http://cbioportal.org/msk-impact; ICB cohort, DOI: 10.1126/science.aao4572. The raw sequence data reported in this paper have been deposited in the Genome Sequence Archive in National Genomics Data Center under accession number HRA000456 that are publicly accessible at: http://bigd.big.ac.cn/gsa-human. All other data are available from the corresponding authors upon reasonable request.

## Provenance and peer review

Not commissioned, externally peer-reviewed.

## Supplementary Material
