# Supplementary material for: Specific human leukocyte antigen class I genotypes predict prognosis in resected pancreatic adenocarcinoma: a retrospective cohort study
Source: Int J Surg. 2023 Apr 7;109(7):1941–52. doi: 10.1097/JS9.0000000000000264 (PMC10389500; doi:10.1097/JS9.0000000000000264)
Supplement: Supplementary file 3 [file js9-109-1941-s003.pdf]

Supplementary content

Supplementary Methods

Workflow of next-generation sequencing

Briefly, genomic DNA of tumor tissues was extracted using TIANamp Genomic DNA Kit (TIANGEN, China) and normal DNA of peripheral blood cells was extracted using TGuide S32 Magnetic Blood Genomic DNA Kit (TIANGEN, China). The concentration of isolated DNA was determined by Qubit dsDNA HS (High Sensitivity) Assay Kit (Themo Fisher, USA), while the quality of DNA was evaluated by Agilent 2100 BioAnalyzer (Agilent, USA). Captures of targeted regions were performed using HyperCap Target Enrichment Kit (Roche, Swiss). Hybridization and washing were conducted according to the manufacturer's protocol. The captured library was sequenced on the instruments of Illumina Novaseq 6000 or NextSeq CN500 producing paired-end reads with the length of each end as 150bp. The average deduped sequencing depth of tissues and blood cells were 830× and 240× respectively. After data quality control via Trimmomatic (v0.36)[1], reference mapping (hg19) by BWA aligner (v0.7.17)[2] as well as duplication masking by Picard (v2.23.0), processed BAM files were obtained and used for the following analyses. Next, processed BAM files were imported into software VarDict (v1.5.1)[3] and FreeBayes (v1.2.0)[4] for SNVs, INDELs and complex mutations calling. All variants were annotated by using ANNOVAR[5] and filtered according to the below criteria: i) VAF ≥ 2%, total support reads ≥6, and without strand bias; ii) in exonic region and annotated as nonsynonymous variants; iii) allele frequency ≤ 0.002 in both the Exome Aggregation Consortium (ExAC) database[6] and the Genome Aggregation Database (gnomAD, MacArthur Lab). CNV was analyzed by CNVkit (v0.9.2)[7] and the cutoff value of CNV gain is copy number >=3 and the cutoff value of CNV loss is copy number <=1.2.

References

1. Bolger AM, Lohse M, Usadel B. Trimmomatic: a flexible trimmer for Illumina sequence data. Bioinformatics 2014; 30: 2114-2120.
2. Li H, Durbin R. Fast and accurate short read alignment with Burrows-Wheeler transform. Bioinformatics 2009; 25: 1754-1760.
3. Lai Z, Markovets A, Ahdesmaki M et al. VarDict: a novel and versatile variant caller for next-generation sequencing in cancer research. Nucleic Acids Res 2016; 44: e108.
4. Garrison E, Marth G. Haplotype-based variant detection from short-read sequencing. arXiv preprint arXiv:1207.3907 2012.
5. Wang K, Li M, Hakonarson H. ANNOVAR: functional annotation of genetic variants from high-throughput sequencing data. Nucleic Acids Res 2010; 38: e164.
6. Karczewski KJ, Weisburd B, Thomas B et al. The ExAC browser: displaying reference data information from over 60 000 exomes. Nucleic Acids Res 2017; 45: D840-D845.
7. Talevich E, Shain AH, Botton T, Bastian BC. CNVkit: Genome-Wide Copy Number Detection and Visualization from Targeted DNA Sequencing. PLoS Comput Biol 2016; 12: e1004873.

Supplementary Figures

Figure S1 The prognostic role of specific *HLA*-I genotypes in Chinese resected PAAD patients characterized by clinical stages or CA 19-9. (**A**) Unsupervised clustering of HLA-A/B supertypes in 226 resected PAAD patients. (**B**) Kaplan-Meier analyses of DFS by HLA-A02^{+}B62^{+}B44^{-} in stage III PAAD patients (n=41) undergoing radical resections. *P* value was calculated by two-sided Log-rank test. (**C**, **D**) Kaplan-Meier analyses of DFS by HLA-B62/B44 supertype combinations in A02-postive PAAD individuals (n=125) (**C**) or stage I-II individuals with A02 supertype (n=103) (**D**). *P* value was calculated by two-sided Log-rank test. (**E**) Association of B44 supertype with extended DFS in extremely early-stage (IA-IB) individuals (n=86). *P* value was calculated by two-sided Log-rank test. (**F**) Kaplan-Meier analyses of DFS by HLA-A02^{+}B62^{+}B44^{-} in high-CA 19-9 (> 37 U/ml) PAAD patients (n=158) undergoing radical resections. *P* value was calculated by two-sided Log-rank test. (**G**) comparison of postsurgical relapse rates within 6 months among the stage I-II individuals characterized by HLA-A02^{+}B62^{+}B44^{-} or not and high/low level (> or ≤37 U/ml) of CA 19-9. *P* value was calculated by two-sided Chi-square test. $P < 0.05$ denoted statistical significance. TNM staging, AJCC 8th edition.

Figure S2 Multivariable Cox proportional-hazards regression analysis of HLA-A02^{+}B62^{+}B44^{-} impacting DFS in stage III PAAD patients (n=41). WT, wild type; Mut, mutant. $P < 0.05$ denoted statistical significance. Bars represent the 95% confidence interval (CI). TNM staging, AJCC 8th edition.

Figure S3 Multivariable Cox proportional-hazards regression analysis of HLA-A02^{+}B62^{+}B44^{-} impacting DFS in low-CA 19-9 (≤37 U/ml) PAAD patients (n=68). WT, wild type; Mut, mutant. $P < 0.05$ denoted statistical significance. Bars represent the 95% confidence interval (CI). TNM staging, AJCC 8th edition.

Figure S4 Multivariable Cox proportional-hazards regression analysis of HLA-A02^{+}B62^{+}B44^{-} impacting DFS in high-CA 19-9 (>37 U/ml) PAAD patients (n=158). WT, wild type; Mut, mutant. $P < 0.05$ denoted statistical significance. Bars represent the 95% confidence interval (CI). TNM staging, AJCC 8th edition.

Figure S5 Comparison of *HLA*-I genotype in early-stage PAAD patients with long-term survival or early-recurrence. (**A**) Decrease of the proportions of HLA-A02^{+}B62^{+}B44^{-} and A02 supertype from ER3/6 cohorts towards LTS5/6/7/8 cohorts. ER3/6, earl-recurrence in 3 or 6 months after surgery; LTS5/6/7/8, long-term survival > 5, 6, 7 or 8 years since surgery. *P* value was calculated by one-sided Chi-square test. $P < 0.05$ denoted statistical significance. *, $P < 0.05$, compared with ER3 cohort. (**B**) Frequency distribution of HLA-A02 supertype among ER3 cohort, LTS5-7 cohort and LTS7 cohort. LTS5-7, long-term survival of 5-7 years since surgery. The numerical values denoted in rows were the number of subgroup patients. *P* value was calculated by Chi-square test for trend. $P < 0.05$ denoted statistical significance. TNM staging, AJCC 8th edition.

Figure S6 Association of *HLA*-I genotype with common driver mutations or oncogenic pathway alterations in PAAD patients. (**A**) Spearman rank correlation between HLA-A02/B62/B44 supertype combinations or single supertype and mutations of common driver genes (*KRAS*, *TP53*, *CDKN2A* and *SMAD4*) in 226 PAAD patients undergoing radical resections. (**B**) Spearman rank correlation between HLA-A02/B62/B44 supertype combinations or single supertype and genetic alterations of 10 vital oncogenic pathways in 226 PAAD patients undergoing radical resections. $P < 0.05$ denoted statistical significance; ns, not significant.

Figure S7 Differential gene expression between specific *HLA*-I genotype or not in tumor microenvironment of early-stage PAAD patients. (**A**) Identification of the genes with differential expression through R package limma with *P* value < 0.05 and abs(log_2fold change) > 1 between A02-positive versus A02-negative individuals in stage I-II PAAD patients (n=44) with well/moderate differentiation. (**B**) A significantly lower expression level of *HLA-A* in tumor tissues of the individuals with HLA-A02 supertype versus A02-negative individuals in stage I-II PAAD patients (n=44) with well/moderate differentiation. (**C**) Identification of the genes with differential expression through R package limma with *P* value < 0.05 and abs(log_2fold change) > 1 between A03-positive versus A03-negative individuals in stage I-II PAAD patients (n=44) with well/moderate differentiation. (**D**) A significantly higher expression level of *HLA-A* in tumor tissues of the individuals with HLA-A03 supertype versus A03-negative individuals in stage I-II PAAD patients (n=44) with well/moderate differentiation. (**E**) Identification of the genes with differential expression through R package limma with *P* value < 0.05 and abs(log_2fold change) > 1 between B62-positive versus B62-negative individuals in stage I-II PAAD patients (n=44) with well/moderate differentiation. (**F**) Identification of the genes with differential expression through R package limma with *P* value < 0.05 and abs(log_2fold change) > 1 between B44-negative versus B44-positive individuals in stage I-II PAAD patients (n=44) with well/moderate differentiation. *P* value was calculated by two-sided wilcoxon test. *P* < 0.05 denoted statistical significance. *, *P* < 0.05; **, *P* < 0.01; ***, *P* < 0.001; ns, not significant. Boxplots indicate the median, the first and third quartiles. Bars represent 1.5 times the interquartile range and outlying points are plotted separately. TNM staging, AJCC 8th edition.

Figure S8 Differential expression of immune-related signatures or markers between specific *HLA*-I genotype or not in tumor microenvironment of early-stage PAAD patients. (**A-D**) Comparison of expression levels of T cell-inflamed GEP (**A**), T cell markers (**B**), IFN-γ signature (**C**) and Monocyte (**D**) in tumor tissues of the individuals with HLA-A02$^+$B62$^+$B44$^-$ or single A02/B62/B44 supertype versus other individuals in stage I-II PAAD patients (n=44) with well/moderate differentiation. *P* value was calculated by two-sided wilcoxon test. *P* < 0.05 denoted statistical significance. ns, not significant. Boxplots indicate the median, the first and third quartiles. Bars represent 1.5 times the interquartile range and outlying points are plotted individually. TNM staging, AJCC 8th edition.

Figure S9 Validation of HLA-A02$^+$B62$^+$B44$^-$-correlated driver mutations in public datasets of PAAD. (**A**, **B**) Kaplan-Meier analyses of OS by *KRAS* G12D and *TP53* co-mutations in PAAD patients (*N* = 179) (**A**) or stage I-II PAAD patients (*N* = 167) (**B**) of TCGA. *P* value was calculated by two-sided Log-rank test. (**C**, **D**) Kaplan-Meier analyses of OS by *KRAS* G12D and *TP53* co-mutations in PAAD patients (*N* = 235) (**C**) or stage I-II PAAD patients (*N* = 160) (**D**) of ICGC. *P* value was calculated by two-sided Log-rank test. (**E**) Kaplan-Meier analysis of OS by concurrent *KRAS* G12D & *TP53* mutations in metastatic PAAD patients (*N* = 293). *P* value was calculated by two-sided Log-rank test.

Supplementary Tables

Table S1

Profiles of germline *HLA-A*/*B*/*C* alleles and corresponding supertypes identified in 608 or 226 Chinese PAAD patients.

Table S2

The influence of different HLA-I supertypes on DFS in 226 Chinese PAAD patients.

**HLA-I supertype**	**Proportion (%)**	**HR (95% CI)**	***P* value**
A02	55.31	1.18 (0.80-1.73)	0.4014
B62	37.61	1.19 (0.81-1.74)	0.3845
B44	35.84	0.83 (0.55-1.24)	0.3576
A03	60.18	0.77 (0.52-1.13)	0.1753
B07	34.96	0.90 (0.60-1.34)	0.5968
A24	30.97	0.88 (0.58-1.33)	0.5344
B27	21.24	0.87 (0.54-1.42)	0.5879
A01	12.83	1.16 (0.66-2.04)	0.5952
B58	13.72	0.70 (0.38-1.27)	0.2343
B08	1.77	NA	NA
A01A03	11.95	1.91 (1.15-3.17)	0.011
A01A24	0.88	NA	NA

P value was calculated by two-sided Log-rank test. NA, without analysis for few patients.

Table S3

Univariate analyses of the influence of HLA-A02^{+}B62^{+}B44^{-} or single A02/B62/B44 supertype on DFS in 226 Chinese PAAD patients and subsets. $P < 0.05$ denoted statistical significance. TNM staging, AJCC 8th edition.

Table S4 Characteristics of radically resected PAAD patients with early recurrence in 3 months (ER3) and long-term survivors with PAAD (> 5 years since surgery, LTS5).

**Characteristics**	**ER3, No. (%) (n = 39)**	**LTS5, No. (%) (n = 42)**	***P* value**
HLA-I genotype			
A02+B62+B44-	20 (51.28)	13 (30.95)	0.331
A02+	27 (69.23)	21 (50)	0.078
Age			
> 65 years	18 (46.15)	12 (28.57)	0.102
Gender			
Male	26 (66.67)	25 (59.52)	0.506
Female	13 (33.33)	17 (40.48)	
pT stage			
T1-T2	17 (43.59)	34 (80.95)	**<0.001**
T3	22 (56.41)	8 (19.05)	
pN stage			
N0	20 (51.28)	32 (76.19)	**0.019**
N1	19 (48.72)	10 (23.81)	
Stage			
IA-IB	10 (25.64)	26 (61.9)	**0.001**
IIA	10 (25.64)	5 (11.9)	
IIB	19 (48.72)	11 (26.2)	
Differentiation			
Well/moderate	14 (35.9)	32 (76.19)	**<0.001**
Poor	25 (64.1)	10 (23.81)	
CA 19-9 > 37 U/ml	25 (64.1)	12 (28.6)	0.117
Adjuvant therapy	24 (61.54)	24 (57.14)	0.688

ER3, early recurrence in 3 months; LTS5, long-term survival > 5 years since surgery. All ER3/LTS5 individuals are early-stage (I-II) PAAD patients following radical resection (R0). *P* value was calculated by two-sided Chi-square test. Boldface indicates the *P* values reaching statistical significance.

Figure S1

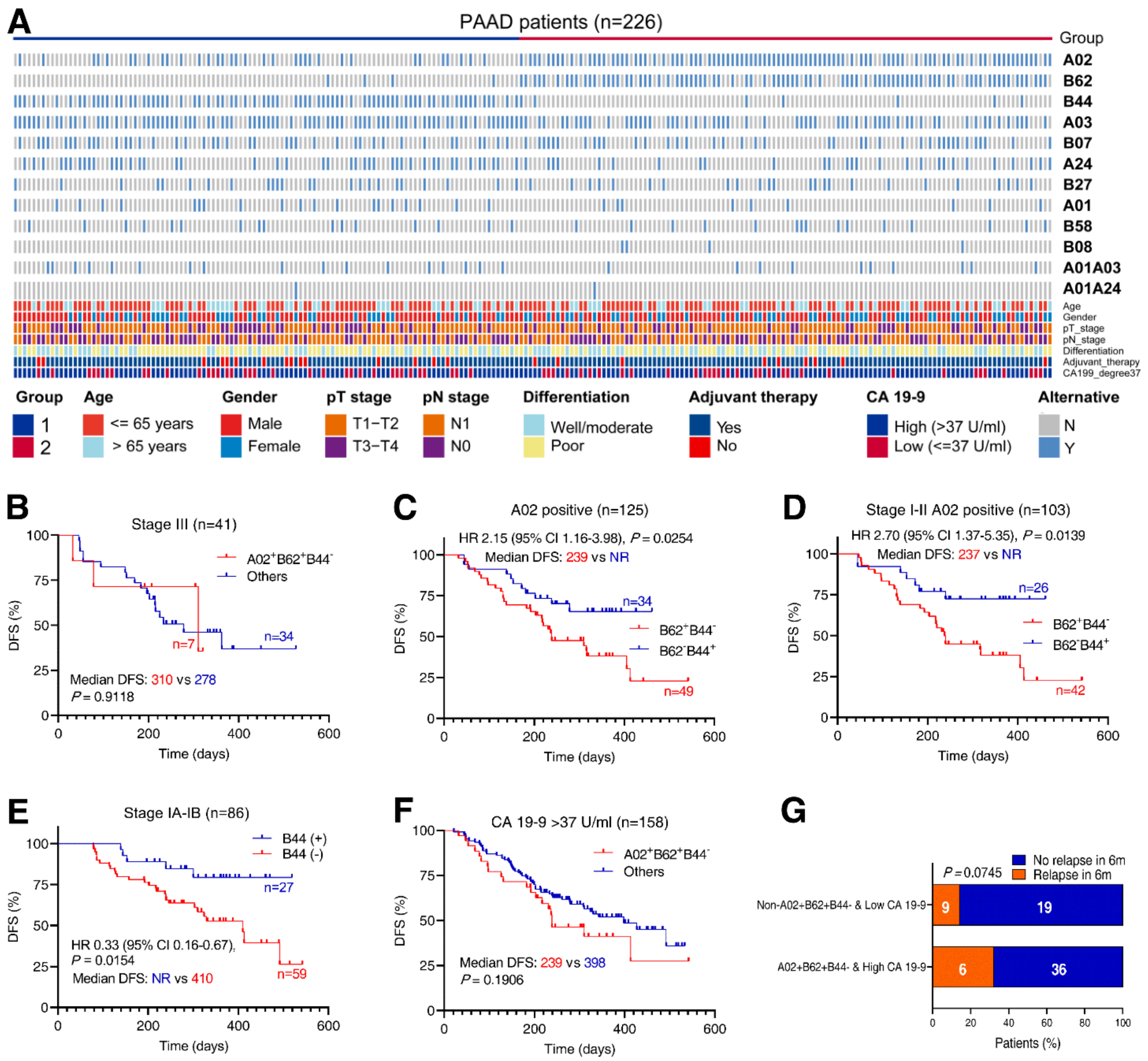

A
PAAD patients (n=226)
Group
A02
B62
B44
A03
B07
A24
B27
A01
B58
B08
A01A03
A01A24
Age
Gender
pT_stage
pN_stage
Differentiation
Adjuvant_therapy
CA199_degree37
Group
1
2
Age
<= 65 years
> 65 years
Gender
Male
Female
pT stage
T1−T2
T3−T4
pN stage
N1
N0
Differentiation
Well/moderate
Poor
Adjuvant therapy
Yes
No
CA 19-9
High (>37 U/ml)
Low (<=37 U/ml)
Alternative
N
Y
B
Stage III (n=41)
A02+B62+B44-
Others
n=7
n=34
Median DFS: 310 vs 278
P = 0.9118
DFS (%)
Time (days)
C
A02 positive (n=125)
HR 2.15 (95% CI 1.16-3.98), P = 0.0254
Median DFS: 239 vs NR
n=34
B62+B44-
B62-B44+
n=49
DFS (%)
Time (days)
D
Stage I-II A02 positive (n=103)
HR 2.70 (95% CI 1.37-5.35), P = 0.0139
Median DFS: 237 vs NR
n=26
B62+B44-
B62-B44+
n=42
DFS (%)
Time (days)
E
Stage IA-IB (n=86)
B44 (+)
B44 (-)
n=27
HR 0.33 (95% CI 0.16-0.67),
P = 0.0154
Median DFS: NR vs 410
n=59
DFS (%)
Time (days)
F
CA 19-9 >37 U/ml (n=158)
A02+B62+B44-
Others
Median DFS: 239 vs 398
P = 0.1906
DFS (%)
Time (days)
G
P = 0.0745
No relapse in 6m
Relapse in 6m
Non-A02+B62+B44- & Low CA 19-9
9
19
A02+B62+B44- & High CA 19-9
6
36
Patients (%)

Figure S2

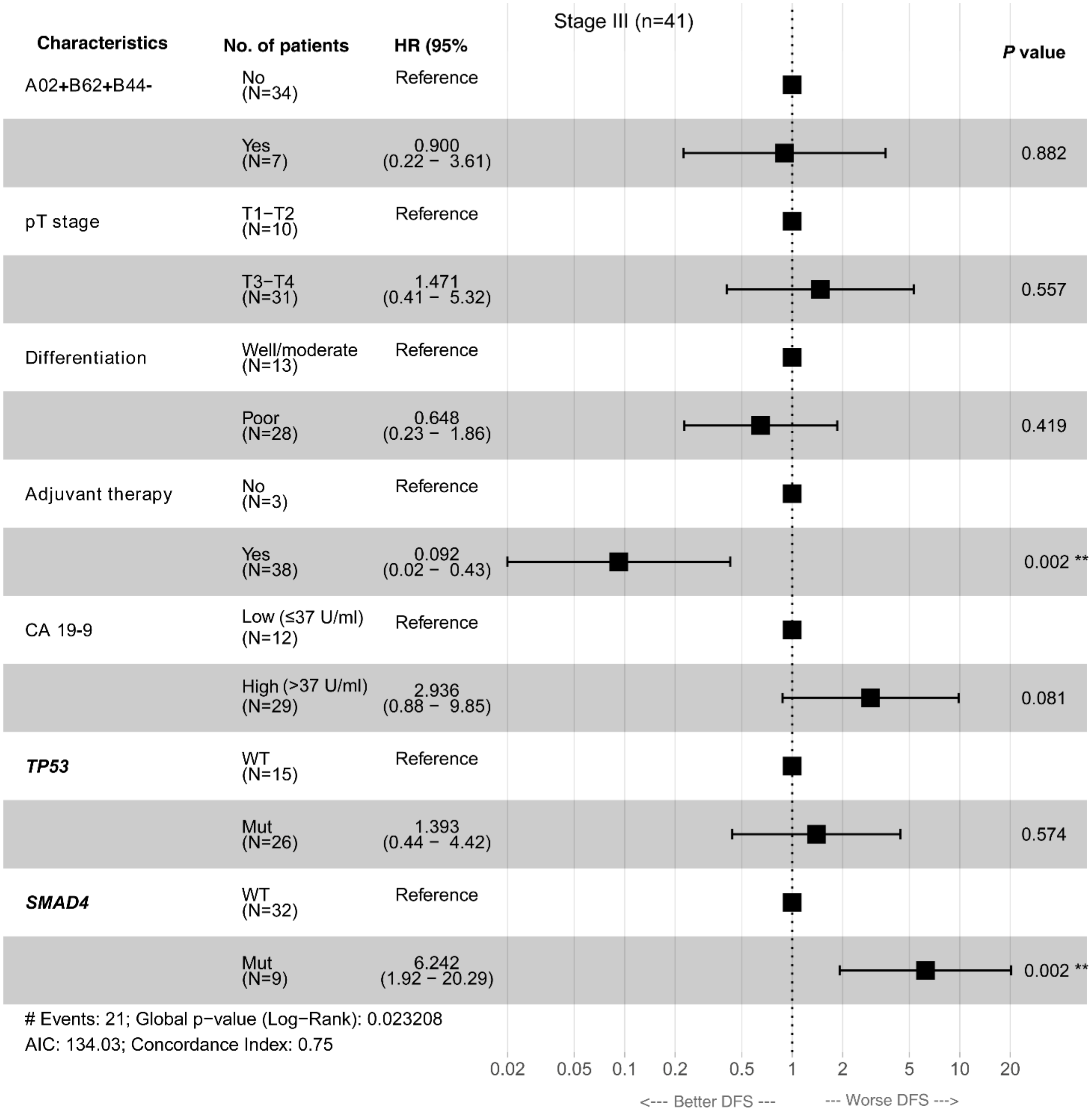

Stage III (n=41)
Characteristics
No. of patients
HR (95%
P value
A02+B62+B44-
No
(N=34)
Reference
Yes
(N=7)
0.900
(0.22 – 3.61)
0.882
pT stage
T1–T2
(N=10)
Reference
T3–T4
(N=31)
1.471
(0.41 – 5.32)
0.557
Differentiation
Well/moderate
(N=13)
Reference
Poor
(N=28)
0.648
(0.23 – 1.86)
0.419
Adjuvant therapy
No
(N=3)
Reference
Yes
(N=38)
0.092
(0.02 – 0.43)
0.002 **
CA 19-9
Low (≤37 U/ml)
(N=12)
Reference
High (>37 U/ml)
(N=29)
2.936
(0.88 – 9.85)
0.081
TP53
WT
(N=15)
Reference
Mut
(N=26)
1.393
(0.44 – 4.42)
0.574
SMAD4
WT
(N=32)
Reference
Mut
(N=9)
6.242
(1.92 – 20.29)
0.002 **
Events: 21; Global p–value (Log–Rank): 0.023208
AIC: 134.03; Concordance Index: 0.75
0.02
0.05
0.1
0.2
0.5
1
2
5
10
20
<--- Better DFS ---
--- Worse DFS --->

Figure S3

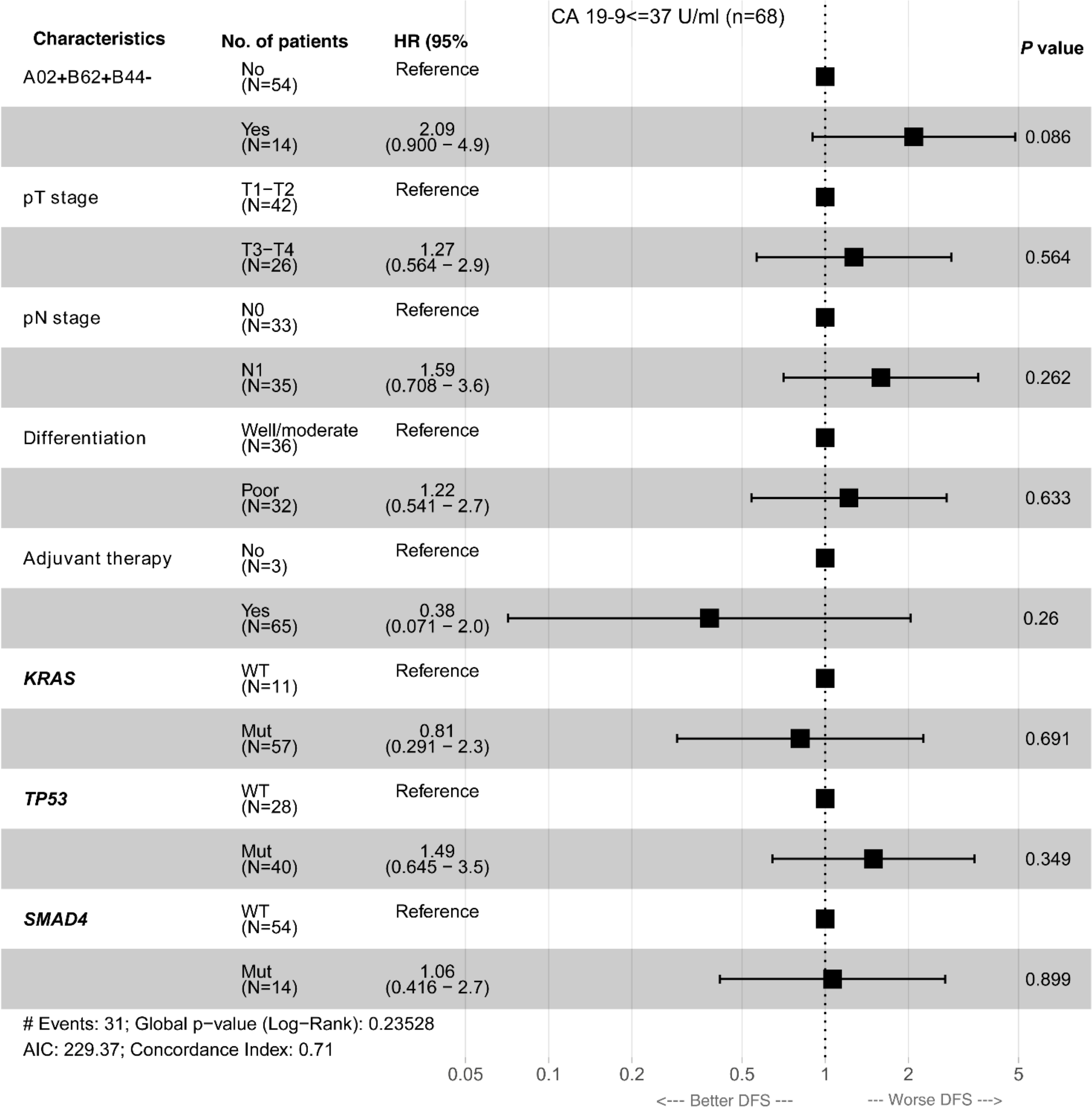
CA 19-9<=37 U/ml (n=68)
Characteristics
No. of patients
HR (95%
P value
A02+B62+B44-
No (N=54)
Reference
Yes (N=14)
2.09 (0.900 − 4.9)
0.086
pT stage
T1−T2 (N=42)
Reference
T3−T4 (N=26)
1.27 (0.564 − 2.9)
0.564
pN stage
N0 (N=33)
Reference
N1 (N=35)
1.59 (0.708 − 3.6)
0.262
Differentiation
Well/moderate (N=36)
Reference
Poor (N=32)
1.22 (0.541 − 2.7)
0.633
Adjuvant therapy
No (N=3)
Reference
Yes (N=65)
0.38 (0.071 − 2.0)
0.26
KRAS
WT (N=11)
Reference
Mut (N=57)
0.81 (0.291 − 2.3)
0.691
TP53
WT (N=28)
Reference
Mut (N=40)
1.49 (0.645 − 3.5)
0.349
SMAD4
WT (N=54)
Reference
Mut (N=14)
1.06 (0.416 − 2.7)
0.899
Events: 31; Global p−value (Log−Rank): 0.23528
AIC: 229.37; Concordance Index: 0.71
0.05
0.1
0.2
0.5
1
2
5
<--- Better DFS ---
--- Worse DFS --->

Figure S4

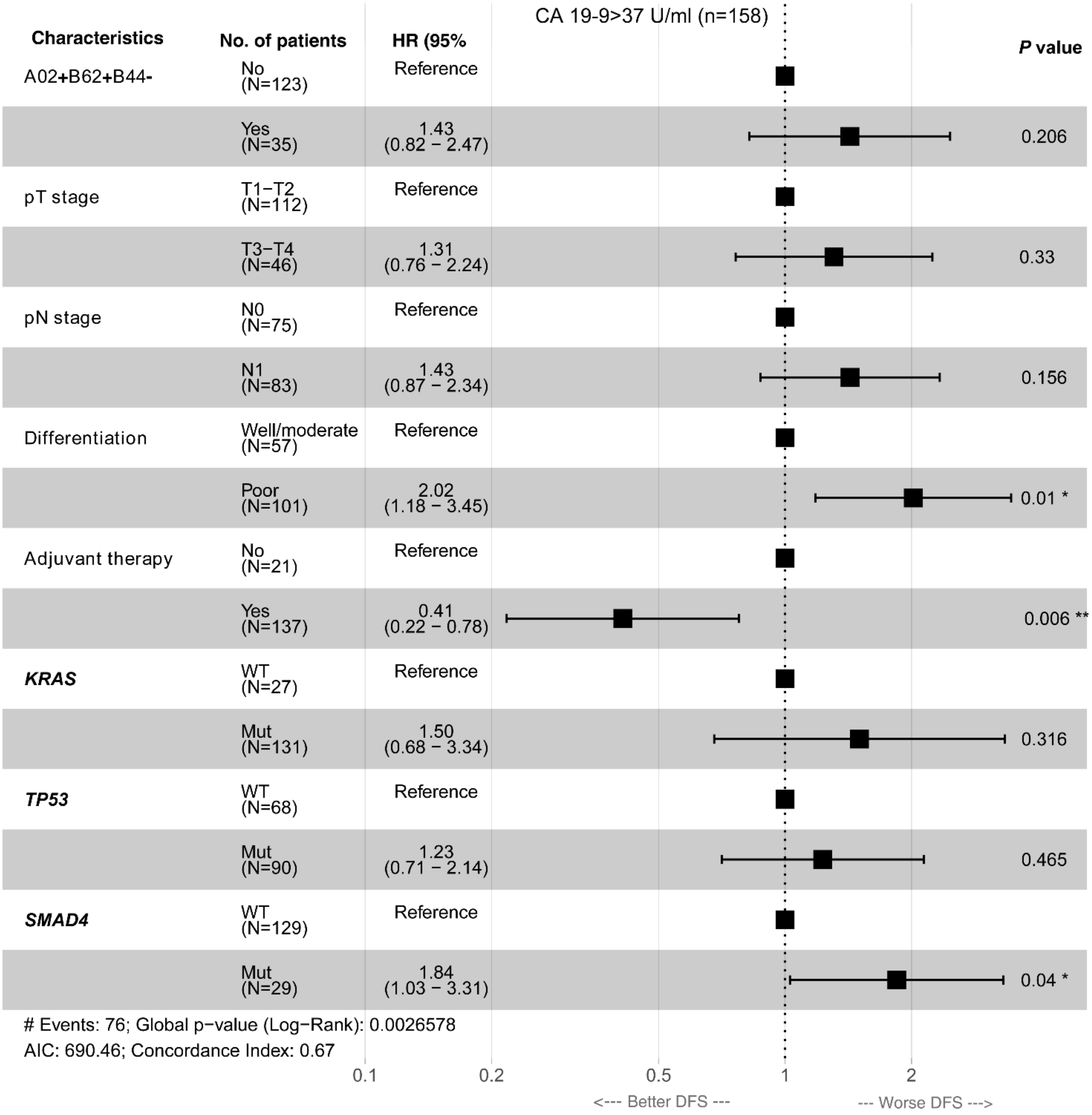
CA 19-9>37 U/ml (n=158)
Characteristics
No. of patients
HR (95%
P value
A02+B62+B44-
No (N=123)
Reference
Yes (N=35)
1.43 (0.82 − 2.47)
0.206
pT stage
T1−T2 (N=112)
Reference
T3−T4 (N=46)
1.31 (0.76 − 2.24)
0.33
pN stage
N0 (N=75)
Reference
N1 (N=83)
1.43 (0.87 − 2.34)
0.156
Differentiation
Well/moderate (N=57)
Reference
Poor (N=101)
2.02 (1.18 − 3.45)
0.01 *
Adjuvant therapy
No (N=21)
Reference
Yes (N=137)
0.41 (0.22 − 0.78)
0.006 **
KRAS
WT (N=27)
Reference
Mut (N=131)
1.50 (0.68 − 3.34)
0.316
TP53
WT (N=68)
Reference
Mut (N=90)
1.23 (0.71 − 2.14)
0.465
SMAD4
WT (N=129)
Reference
Mut (N=29)
1.84 (1.03 − 3.31)
0.04 *
Events: 76; Global p−value (Log−Rank): 0.0026578
AIC: 690.46; Concordance Index: 0.67
0.1
0.2
0.5
1
2
<--- Better DFS ---
--- Worse DFS --->

Figure S5

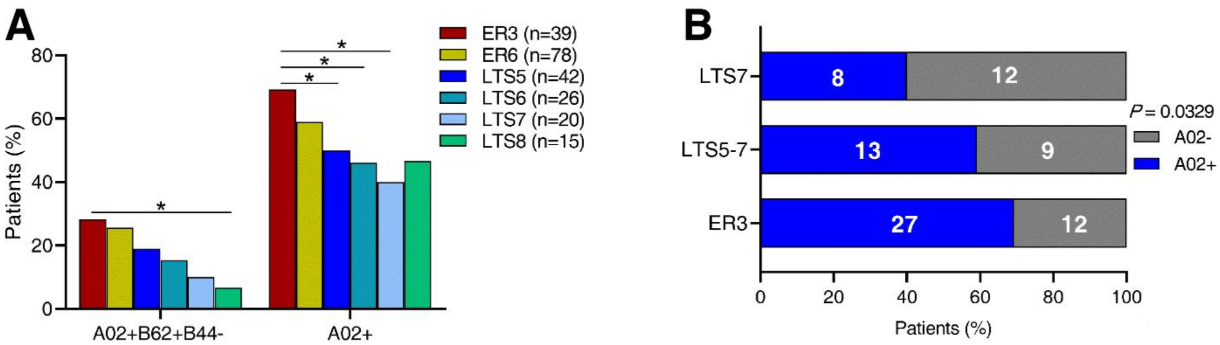
A
B
80
60
40
20
0
Patients (%)
*
A02+B62+B44-
A02+
ER3 (n=39)
ER6 (n=78)
LTS5 (n=42)
LTS6 (n=26)
LTS7 (n=20)
LTS8 (n=15)
LTS7
LTS5-7
ER3
8
12
13
9
27
12
P = 0.0329
A02-
A02+
0
20
40
60
80
100
Patients (%)

Figure S6

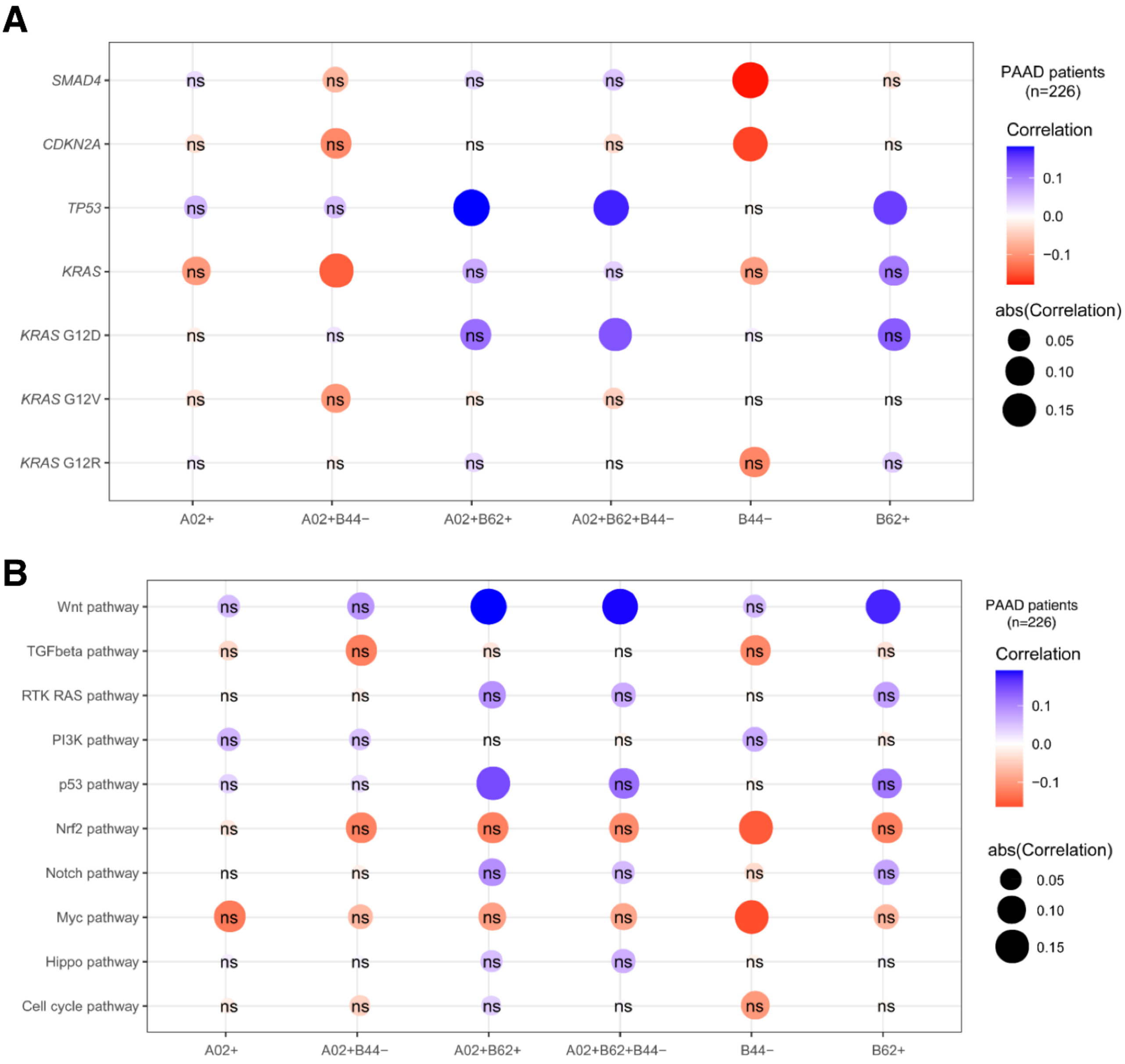
A
SMAD4
CDKN2A
TP53
KRAS
KRAS G12D
KRAS G12V
KRAS G12R
A02+
A02+B44−
A02+B62+
A02+B62+B44−
B44−
B62+
ns
PAAD patients
(n=226)
Correlation
0.1
0.0
−0.1
abs(Correlation)
0.05
0.10
0.15
B
Wnt pathway
TGFbeta pathway
RTK RAS pathway
PI3K pathway
p53 pathway
Nrf2 pathway
Notch pathway
Myc pathway
Hippo pathway
Cell cycle pathway
A02+
A02+B44−
A02+B62+
A02+B62+B44−
B44−
B62+
ns
PAAD patients
(n=226)
Correlation
0.1
0.0
−0.1
abs(Correlation)
0.05
0.10
0.15

Figure S7

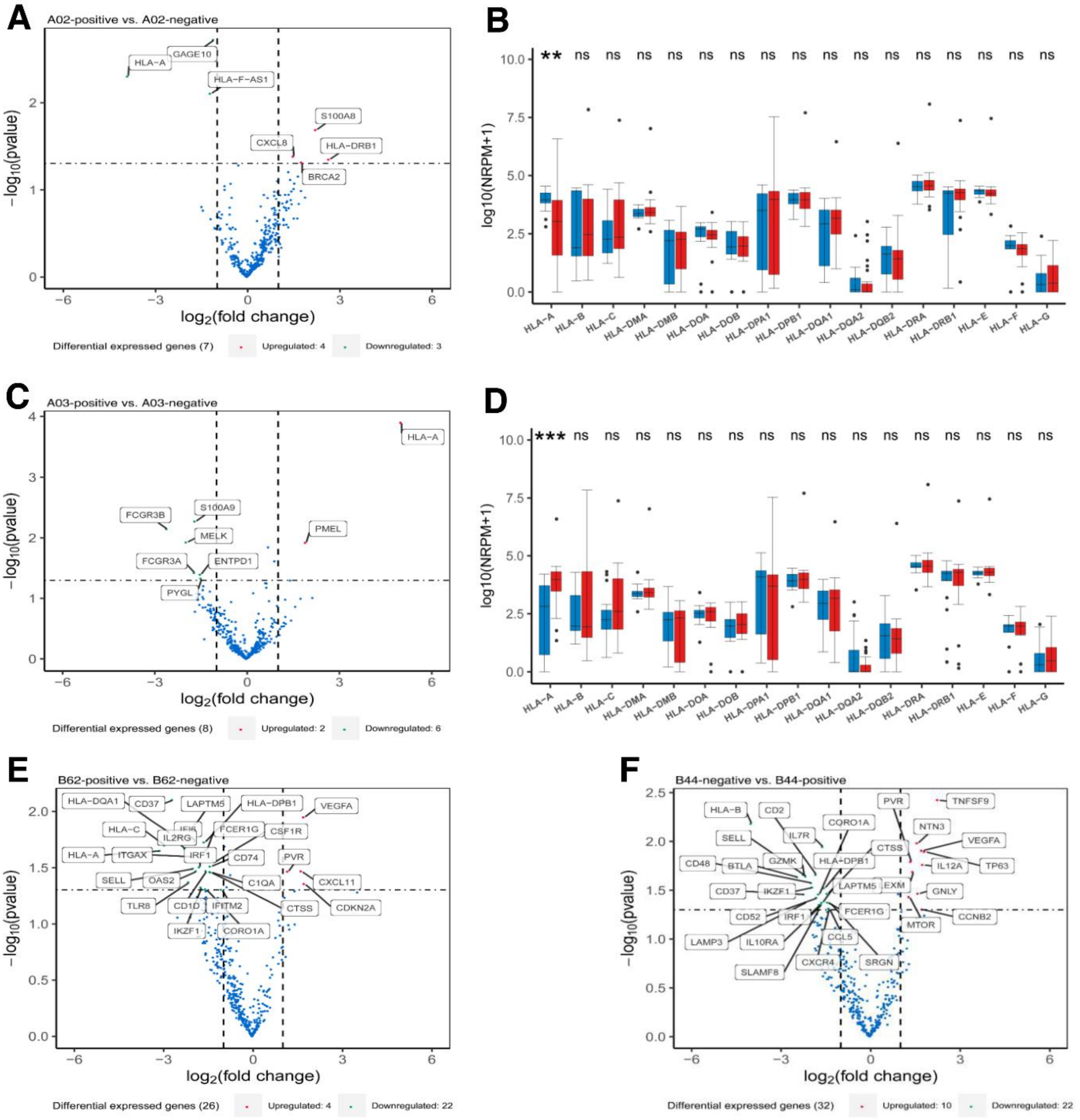
A
A02-positive vs. A02-negative
B
C
A03-positive vs. A03-negative
D
E
B62-positive vs. B62-negative
F
B44-negative vs. B44-positive
log2(fold change)
-log10(pvalue)
log10(NRPM+1)
Subgroup
A02(-)
A02(+)
A03(-)
A03(+)
Differential expressed genes (7) Upregulated: 4 Downregulated: 3
Differential expressed genes (8) Upregulated: 2 Downregulated: 6
Differential expressed genes (26) Upregulated: 4 Downregulated: 22
Differential expressed genes (32) Upregulated: 10 Downregulated: 22

Figure S8

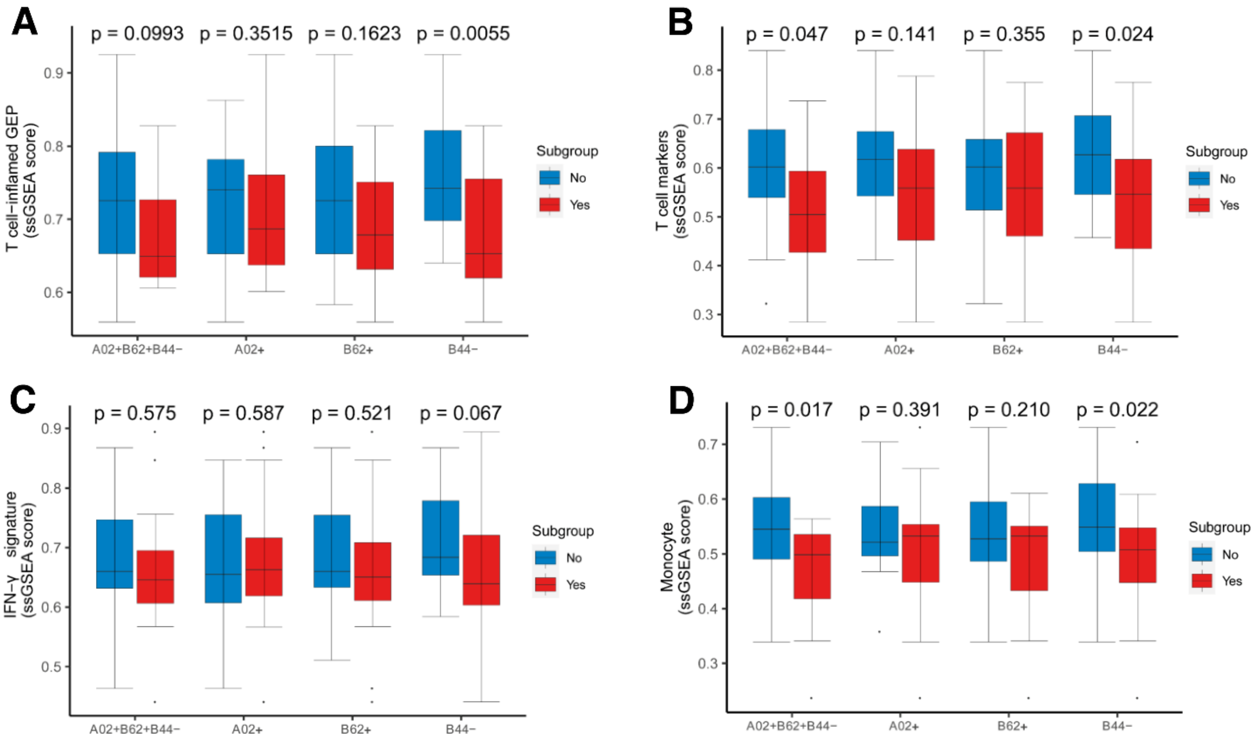
A
p = 0.0993
p = 0.3515
p = 0.1623
p = 0.0055
T cell−inflamed GEP (ssGSEA score)
0.9
0.8
0.7
0.6
A02+B62+B44−
A02+
B62+
B44−
Subgroup
No
Yes
B
p = 0.047
p = 0.141
p = 0.355
p = 0.024
T cell markers (ssGSEA score)
0.8
0.7
0.6
0.5
0.4
0.3
A02+B62+B44−
A02+
B62+
B44−
Subgroup
No
Yes
C
p = 0.575
p = 0.587
p = 0.521
p = 0.067
IFN−γ signature (ssGSEA score)
0.9
0.8
0.7
0.6
0.5
A02+B62+B44−
A02+
B62+
B44−
Subgroup
No
Yes
D
p = 0.017
p = 0.391
p = 0.210
p = 0.022
Monocyte (ssGSEA score)
0.7
0.6
0.5
0.4
0.3
A02+B62+B44−
A02+
B62+
B44−
Subgroup
No
Yes

Figure S9

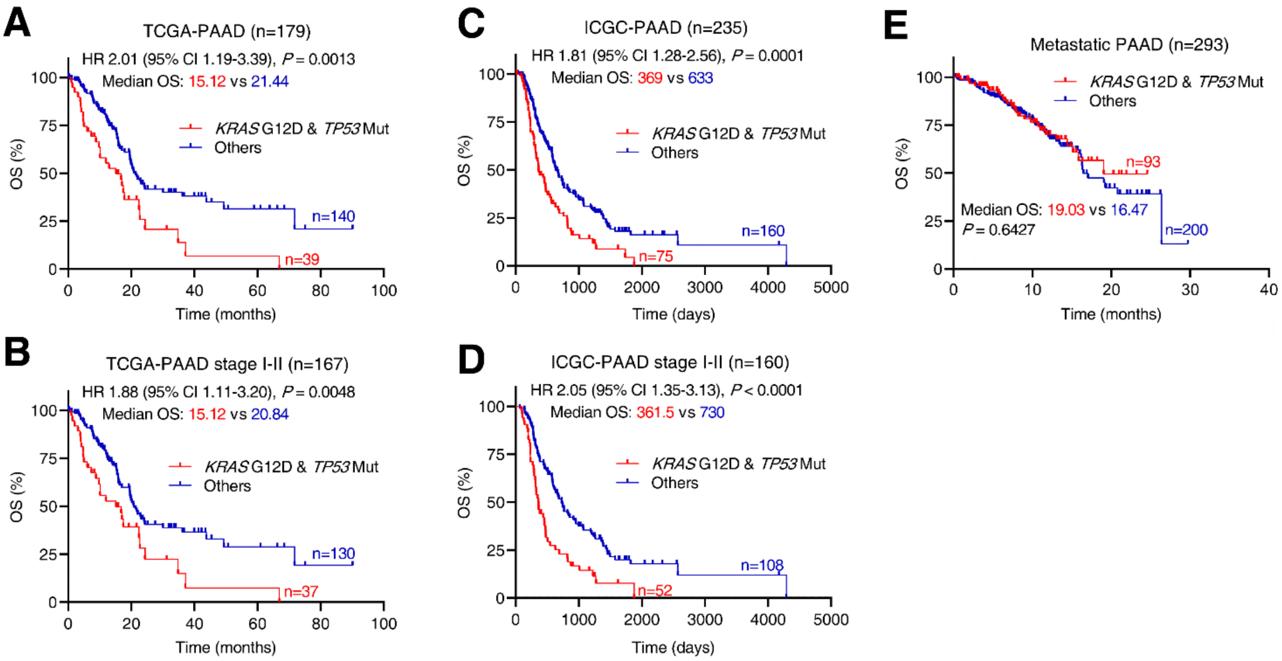

A
TCGA-PAAD (n=179)
HR 2.01 (95% CI 1.19-3.39), P = 0.0013
Median OS: 15.12 vs 21.44
KRAS G12D & TP53 Mut
Others
n=140
n=39
OS (%)
Time (months)
B
TCGA-PAAD stage I-II (n=167)
HR 1.88 (95% CI 1.11-3.20), P = 0.0048
Median OS: 15.12 vs 20.84
KRAS G12D & TP53 Mut
Others
n=130
n=37
OS (%)
Time (months)
C
ICGC-PAAD (n=235)
HR 1.81 (95% CI 1.28-2.56), P = 0.0001
Median OS: 369 vs 633
KRAS G12D & TP53 Mut
Others
n=160
n=75
OS (%)
Time (days)
D
ICGC-PAAD stage I-II (n=160)
HR 2.05 (95% CI 1.35-3.13), P < 0.0001
Median OS: 361.5 vs 730
KRAS G12D & TP53 Mut
Others
n=108
n=52
OS (%)
Time (days)
E
Metastatic PAAD (n=293)
KRAS G12D & TP53 Mut
Others
n=93
n=200
Median OS: 19.03 vs 16.47
P = 0.6427
OS (%)
Time (months)
